# Spatio-temporal analysis of small-area intestinal parasites infections in Ghana

**DOI:** 10.1038/s41598-017-12397-1

**Published:** 2017-09-22

**Authors:** F. B. Osei, A. Stein

**Affiliations:** 1 Department of Mathematics and Statistics, University of Energy and Natural Resources, Sunyani, Ghana; 2 Faculty of Geo-Information Science and Earth Observation (ITC), University of Twente, Enschede, Netherlands

## Abstract

Intestinal parasites infection is a major public health burden in low and middle-income countries. In Ghana, it is amongst the top five morbidities. In order to optimize scarce resources, reliable information on its geographical distribution is needed to guide periodic mass drug administration to populations of high risk. We analyzed district level morbidities of intestinal parasites between 2010 and 2014 using exploratory spatial analysis and geostatistics. We found a significantly positive Moran’s Index of spatial autocorrelation for each year, suggesting that adjoining districts have similar risk levels. Using local Moran’s Index, we found high-high clusters extending towards the Guinea and Sudan Savannah ecological zones, whereas low-low clusters extended within the semi-deciduous forest and transitional ecological zones. Variograms indicated that local and regional scale risk factors modulate the variation of intestinal parasites. Poisson kriging maps showed smoothed spatially varied distribution of intestinal parasites risk. These emphasize the need for a follow-up investigation into the exact determining factors modulating the observed patterns. The findings also underscored the potential of exploratory spatial analysis and geostatistics as tools for visualizing the spatial distribution of small area intestinal worms infections.

## Introduction

Intestinal parasites, helminth and protozoa, are among the most common infections of humans in developing countries with considerable morbidity and substantial burden on public health. The common intestinal parasites, *Ascaris lumbricoides* (roundworms), *Trichuris trichiura* (whipworms), and *Necator americanus or Ancylosttoma duodenale* (hookworms) are estimated to cause one fourth of the known human infectious diseases^1^. Although there are concerns about the actual numbers of infections^2^, intestinal parasites are estimated to infect more than 1 billion people^3,4^. In children, infections can retard child growth, cause anemia, and create cognitive and physical challenges^5–7^. Prevalence is high amongst resource-poor countries, especially in Asia, Sub-Sharan Africa, and Latin America where there is limited access to water supply and poor sanitation. Chemotherapy based on Mass Drug Administration (MDA) is the contemporary control strategy embraced by the World Health Organization (WHO) and governments to reduce infections. The recommendation is to ensure periodic administration of albendazole and mebendazole to at-risk populations^3,8^. To ensure that MDA control programs are focused appropriately on efficient utilization of scarce resources, targeting of populations requiring intervention is essential. This requires an understanding of the nature of spatial patterns, and precise estimates of the local risks for comparison.

Ghana is among the Sub-Saharan countries with high prevalence of intestinal parasites infections^3,4^. The disease has constantly been listed amongst the top five outpatient morbidities. Prevalence has been reported to be between 2% and 78% for various parasites and specific population groups^9–14^. Using random sampling, Ayeh-Kumi *et al*.^10^ estimated the prevalence of intestinal parasites among food vendors in Accra Metropolitan area to be 21.6%, with helminthic predominating with 15.2%. The prevalence of hookworm infection among school-aged children was estimated to be 39.1% in the Kintampo North Municipality, with significant risk factors being age, malaria parasitemia, lack of health care, school area, levels of antibodies against hookworm, and low consumption of animal foods. Asymptomatic carriage among psychiatric patients was estimated to be 13.5%^13^ among some Ghanaian orphanages. These studies focused on either the biological or anthropogenic characteristics of the individuals affected. Besides, prevalences have either been estimated for single geographic units or among specific population categories, hence are unable evaluate the spatial patterns of infection. Intestinal parasites thrive under climatic and environmental conditions such as warm temperatures, high precipitation and adequate soil moisture^15–17^. Infections have often been associated with sociodemographic conditions such as poverty, poor sanitation, and poor drinking water^18–20^. In Ghana, prevalence has been associated with sociodemographic conditions^14^. Since these underlying risk factors are spatially dependent, morbidity rates will be expected to exhibit spatially dependent patterns.

Spatial analysis and geostatistics can provide opportunities to study the type and nature of spatial patterns, and where these patterns occur. They have widely been used to study the spatial patterns and estimate the spatial risk of intestinal parasites infections^17,21–25^. Spatial analysis methods such the global Moran’s Index^26^ (Moran’s *I* hereafter) and its local counterpart, Anselin’s Local Indicator for Spatial Association (LISA)^27^, could illuminate potential causal factors of diseases^28,29^. Geostatistical analysis of health outcomes has also recently received increasing attention as a filtering tool^30–32^. For instance, Poisson kriging allows filtering of those noise by integrating population heterogeneities to account for non-constant variance^30^.

In this paper, we utilize spatial analysis tools and geostatistics to study the spatial patterns and provide spatially explicit maps of risk estimates useful for guiding control programs. Our specific objectives are to (1) quantify the type of spatial association, and detect and map clusters, (2) quantify the nature of spatial structure and map risk estimates of intestinal parasites using district level morbidities in Ghana. As neighborhood health planning in Ghana is largely based upon small-areas (administrative districts), studying spatial patterns of infections at the district level will present valuable and easy to implement information.

## Methods

### Study area and data

#### Study Area and Data

**Figure 1:**
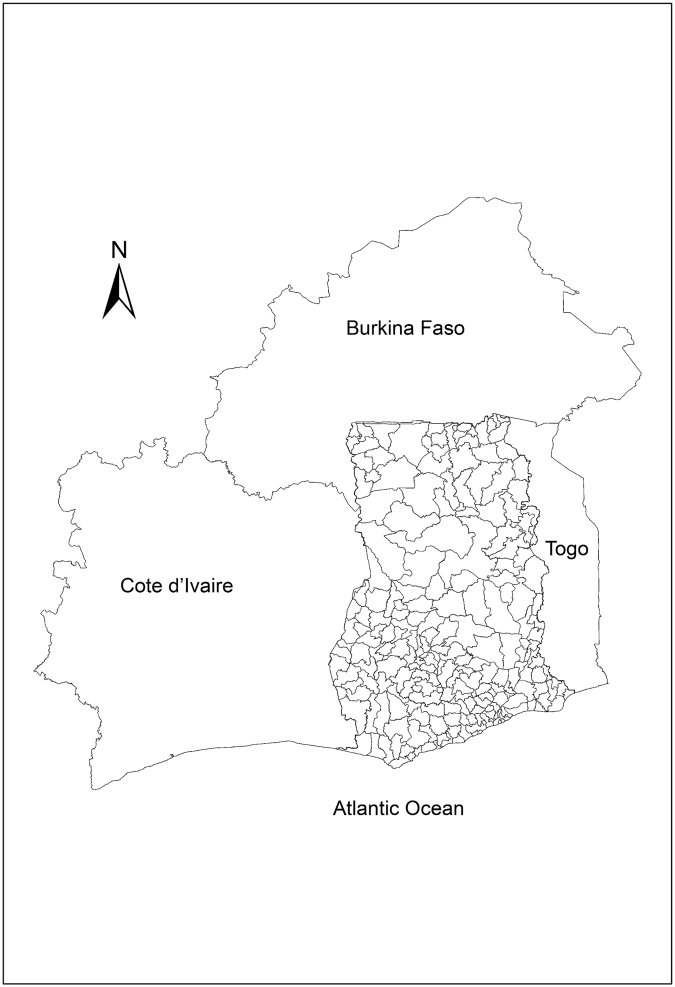
District map of Ghana showing its neighboring countries; Cote d’Ivaire (left), Burkina Faso (top) and Togo (right). This map was created using ArcGIS software (version 10.1, ESRI Inc. Redlands, CA, USA. https://www.esri.com/).

**Figure 2:**
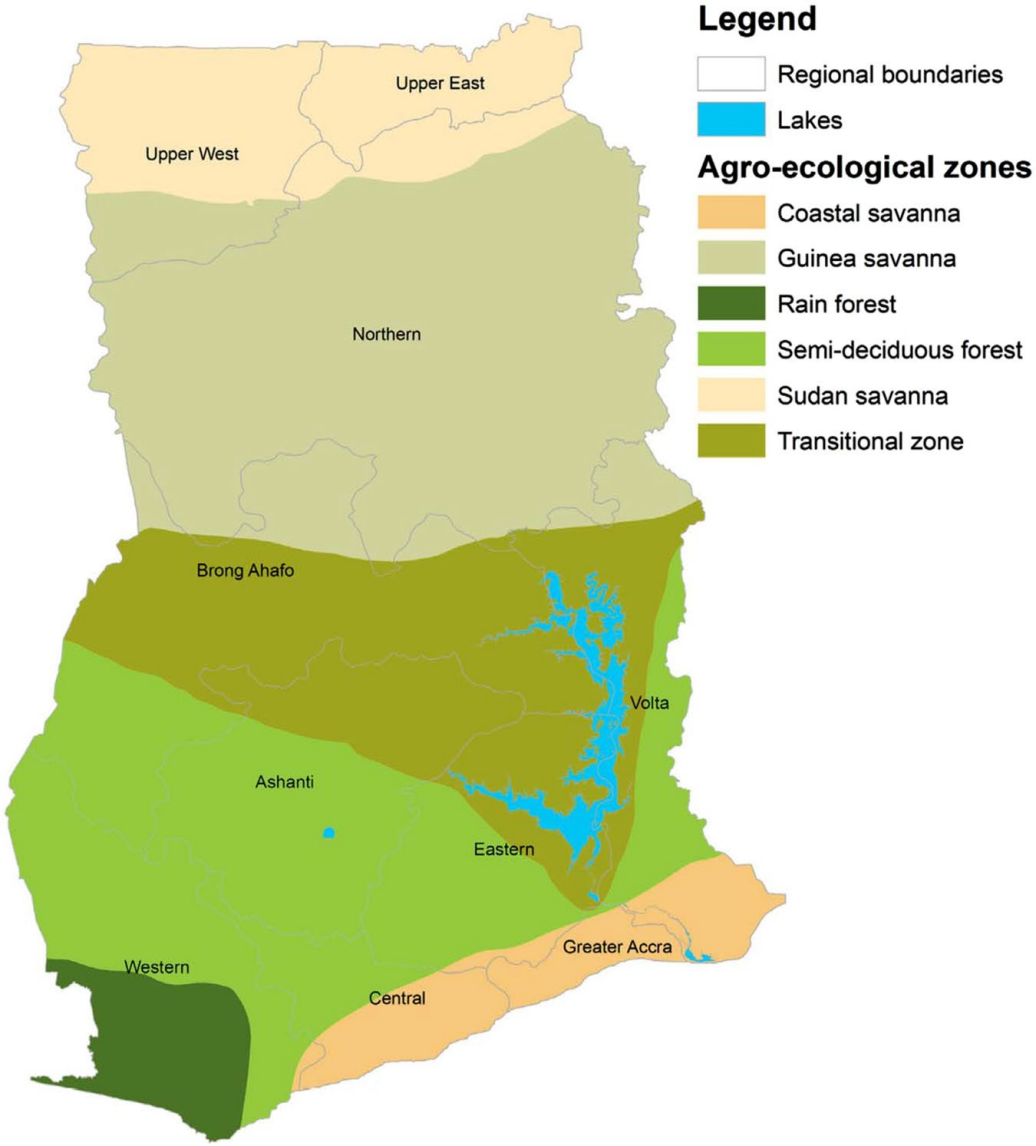
Map showing the six agro-ecological zones of Ghana. This map was created using ArcGIS software (version 10.1, ESRI Inc. Redlands, CA, USA. https://www.esri.com/).

Ghana is a tropical region centrally located on the West Coast of Africa with a total land area of 239,000 km^2^ (Fig. 1: created with ArcGIS software). The average annual temperature is approximately 26 °C (79 °F). There are two distinct rainy seasons, April-June and September-November, but March-September for the northern belt. Annual rainfall ranges from 1,015 mm in the north to 2,030 mm in the southwest (PHC, 2010). The country consists of ten administrative regions which are subdivided into 216 districts. Ghana is subdivided into six agro-ecological zones: Sudan Savannah, Guinea Savannah, Coastal Savannah, Forest/Savannah transitional zone, Deciduous Forest zone and the Rain Forest zone (Fig. 2).

In this study, we used aggregated clinically or laboratory diagnosed cases of intestinal worms parasites. Due to the protection of patient privacy and perhaps deficiencies in address geocoding systems, publicly available data on precise locations of disease cases are uncommon. Consequently, the spatial scale of our study was limited to the 170 administrative districts of which data were available. We obtained district level yearly aggregated cases of intestinal parasites infections from 2010 to 2014 from the Centre for Health Information and Management (CHIM) of the Ghana Health Service (GHS). CHIM is responsible for compiling and ensuring uniformity in reporting and managing all morbidities reported to health facilities (clinics and polyclinics, hospitals). In summary, health facilities capture and aggregate data and submit to sub-districts. Sub-districts aggregate facility summary reports and submit those to districts. Districts then receive both facility and sub-district summary reports for validation. At each stage of the data recording hierarchy, data can be entered directly into the District Health Information Management System (DHIMS). We also obtained population estimates for 2010 to 2014 from the Ghana Statistical Service (GSS).

### Spatial autocorrelation

We used both global and local Moran’s *I* of spatial autocorrelation to estimate the strength of spatial correlation. The global Moran’s *I* ^26^ estimates the general strength of spatial autocorrelation among districts while its local equivalent, LISA^27^, estimates the spatial autocorrelation between districts and their neighboring districts. Thus, the local Moran’s *I* identifies districts with high and low risks as well spatial outliers. For the observed counts ${y}_{i}$ and populations ${n}_{i}$ for the set of districts $i=1,\mathrm{...},m$, we assumed that the counts are realizations from the Poisson distribution ${Y}_{i}|{r}_{i} \sim {\rm{Poisson}}({n}_{i}\cdot {r}_{i})$. The variable of interest is the risk r_*i*_; its maximum likelihood estimate equals ${r}_{i}={y}_{i}/{n}_{i}$, and its variance ${v}_{i}\propto {n}_{i}^{-1}$. Since the variance is inversely proportional to the population sizes, the required assumption of constant variance is violated and could lead to misleading results of large variances for regions with small populations. We used the empirical Bayesian standardization to account for the unequal variances arising from unequal populations^33^. To do so, we constructed a standardized variate ${r}_{i}^{EB}=({r}_{i}-\bar{r})/\sqrt{{v}_{i}}$ based upon the unconditional marginal expectation $\bar{r}$ and district specific variance ${v}_{i}={s}^{2}+\bar{r}/{n}_{i}$. The method of moments estimates^34,35^ for the mean and variance equals $\bar{r}={\rm{\Sigma }}y/{\rm{\Sigma }}n$ and ${s}^{2}={\sigma }^{2}-\bar{r}/({\rm{\Sigma }}{n}_{i}/m)$, respectively, where ${\sigma }^{2}={\rm{\Sigma }}{n}_{i}{({r}_{i}-\bar{r})}^{2}/{\rm{\Sigma }}{n}_{i}$.

We computed the global Moran’s *I*, $I={{\rm{\Sigma }}}_{i}{{\rm{\Sigma }}}_{j}{w}_{ij}{r}_{i}^{EB}{r}_{j}^{EB}$, and local Moran’s *I*, ${I}_{i}={r}_{i}^{EB}{{\rm{\Sigma }}}_{j}{w}_{ij}{r}_{j}^{EB}$ using the standardized variable ${z}_{i}$. We defined the binary connectivity weight matrix ${w}_{ij}$ as ${w}_{ij}=1$ if $(i)\cap (j)\ne null$, and 0 otherwise, where $(i)$ and $(j)$ are the set of boundary points of district *i* and *j*, respectively. The *m* by *m* weight matrix ${w}_{ij}$ was row-standardized, and satisfied the following conditions (1) symmetry, i.e., ${w}_{ij}={w}_{ji}$, (2) zero diagonal elements, i.e. ${w}_{ii}=0$, and (3) normalization, i.e., ${{\rm{\Sigma }}}_{i}{w}_{ij}=1$. For the local index, ${I}_{i}$, the summation over *j* implies that only the set of neighbors ${J}_{i}$ of *i*, $j\in {J}_{i}$, was included.

To test the null hypothesis of no spatial autocorrelation, we generated 999 independent permutations of the vector $({z}_{1},\mathrm{...},{z}_{m})$ and computed $I$ and ${I}_{i}$ for each permutated vector to generate the empirical distribution. The *p*-value was estimated as the proportion of the number of times the index from the permuted data exceeds the Index from the actual data. We used the *spdep* ^36^ package of the R statistical software^37^ for estimating both global and local Moran’s *I*.

### Spatial structure and smoothing

We used geostatistical smoothing to filter out the noise caused by heterogeneous population distribution. Unlike deterministic smoothers^38^, geostatistical smoothing can account for the range of spatial correlation and estimate the associated uncertainties. We assumed that the risks ${r}_{i}$ are realizations from a second-order stationary random field. Poisson kriging was used to estimate the risk over a given district ${i}_{0}$ as linear combinations of the risk observed for that district ${r}_{i}$, and its neighboring districts ${\hat{r}}_{{i}_{0}}=\hat{\mu }+{C}_{i0}{C}_{ij}^{-1}({r}_{i}-\hat{\mu }{1}_{m})$. The assumption of stationarity implies the spatial mean of the prediction locations ${\hat{\mu }}_{{i}_{0}}$ is the same as the spatial mean of the random variable ${r}_{i}$, $E({r}_{i})={\hat{\mu }}_{{i}_{0}}={\hat{\mu }}_{i}$. Under minimum variance, the best linear unbiased estimate of the spatial mean equals ${({{\rm{1}}^{\prime} }_{m}{C}_{ij}^{-1}{{\rm{1}}}_{m})}^{-1}{{\rm{1}}^{\prime} }_{m}{C}_{ij}{r}_{i}$. We refer to the vector $({r}_{i}-\,\hat{\mu }{1}_{m})$ as the predictor variables, *C*_*i*0_ as a covariance vector between the prediction location and the predictor variable, and *C*_*ij*_ as the covariance matrix of the predictor variables. Essentially, ${C}_{i0}{C}_{ij}^{-1}={\lambda }_{i0}$ yields the so-called kriging weights. We used the variogram model $\hat{\gamma }(h)$ as a structural tool to estimate the covariance function based on the relation ${C}_{ij}={C}_{ii}-\hat{\gamma }(h)$, where *C*_*ii*_ is the variance of the risk or covariance at lag 0.

We used the empirical variogram estimator $\gamma (h)={\{2\Sigma {w}_{ij}\}}^{-1}{\rm{\Sigma }}\{{w}_{ij}{({r}_{i}-{r}_{j})}^{2}-{r}^{\ast }\}$, where $h=|i-j|$ and $N(h)$ is the number of observation pairs separated by the distance *h* between the centroids of districts *i* and *j*. Here, the variogram $\gamma (h)$ depends only on the distance between the centroids of districts, and this refers to the assumption of uniform population density within each district. This is an adjusted experimental variogram estimator proposed by^39,40^, and generalized by Goovaerts^30^ for disease mapping to account for heterogeneous populations. The rate differences $({r}_{i}-{r}_{j})$ are weighted by their corresponding populations ${w}_{ij}=\tfrac{{n}_{i}\cdot {n}_{j}}{{n}_{i}+{n}_{j}}$ to homogenize their variances, where ${r}^{\ast }={\rm{\Sigma }}{n}_{i}{r}_{i}/{\rm{\Sigma }}{n}_{i}$ is the population-weighted mean.

To explore both local ${\gamma }_{{\rm{loc}}}(h)$ and regional ${\gamma }_{{\rm{reg}}}(h)$ level spatial correlations, we fitted permissible nested variogram models $\hat{\gamma }(h)={\hat{\gamma }}_{{\rm{loc}}}(h)+{\hat{\gamma }}_{{\rm{reg}}}(h)$ to $\gamma (h)$ ^41–43^. This implies that the risk with mean *μ* was decomposed as the sum of local ${r}_{i,{\rm{loc}}}$ and regional ${R}_{i,{\rm{reg}}}$ orthogonal random functions, ${r}_{i}={r}_{i,{\rm{loc}}}+{r}_{i,{\rm{reg}}}+\mu$, each with its particular contributory variogram ${\hat{\gamma }}_{{\rm{loc}}}(h)$ and ${\hat{\gamma }}_{{\rm{reg}}}(h)$, respectively. This is useful to unravel scale-dependent spatial autocorrelation patterns. The commonly used variogram models such as the exponential and spherical models have been described elsewhere^43,44^. We fitted local and regional scale nested spherical models ${\hat{\gamma }}_{{\rm{loc}}}(h)$ and ${\hat{\gamma }}_{{\rm{reg}}}(h)$, respectively, using weighted least squares with weights ${w}_{\gamma }(h)=\sqrt{N(h)}/\gamma (h)$. In this paper, the variogram modeling was conducted using the public-domain software poisson_kriging.exe^30^.

## Results

### Spatial autocorrelation

**Figure 3:**
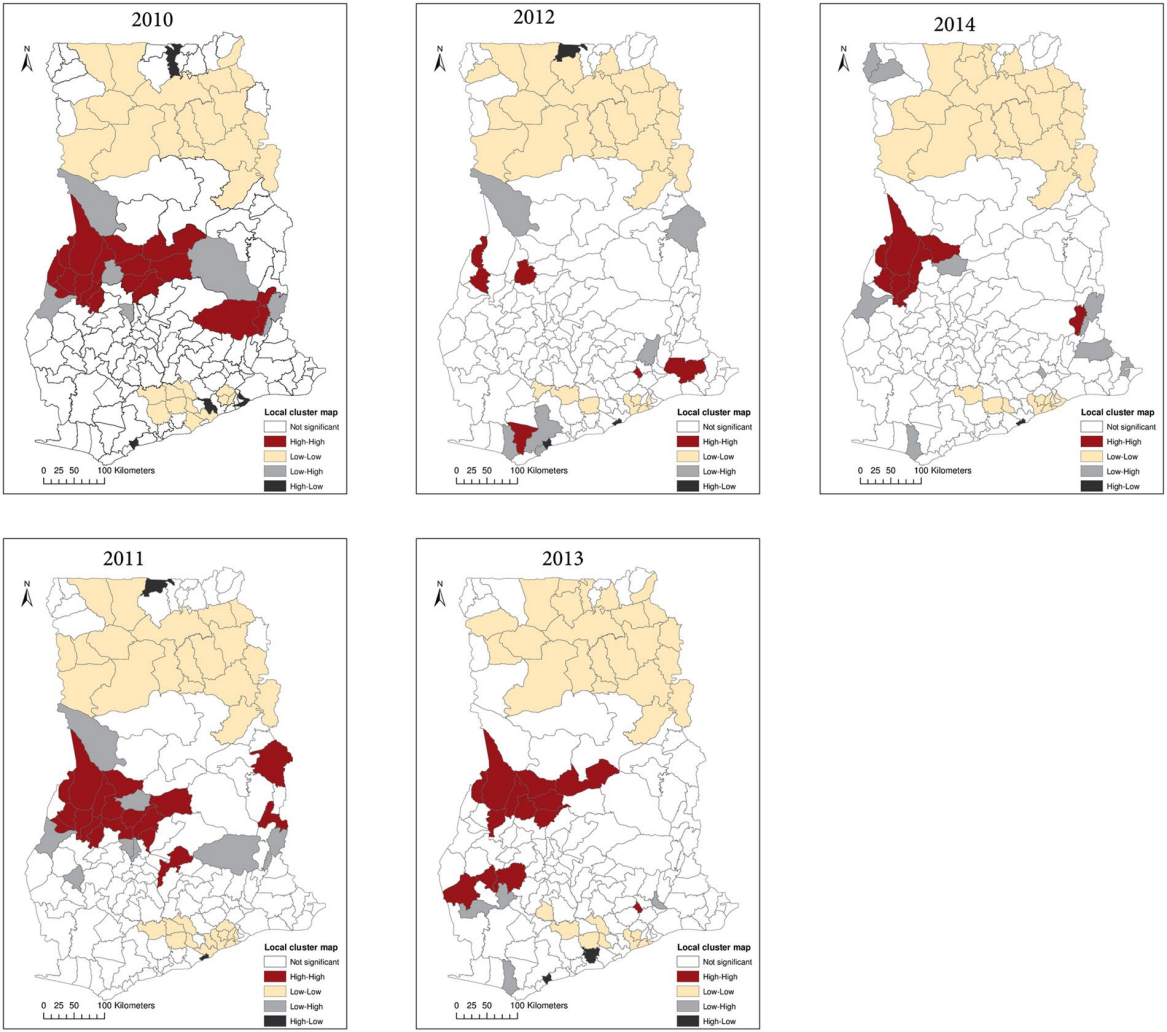
Local Moran’s I cluster maps showing high-high, low-low, low-high, and high-low spatial associations. This map was created using ArcGIS software (version 10.1, ESRI Inc. Redlands, CA, USA. https://www.esri.com/).

Between 2010 and 2014, a total of 3,310,653 intestinal parasites infections were reported. The annual incidence rates ranged from 1.55% to 3.3%, with an average annual incidence rate of 2.53% (Table 1). The incidence rate increased from 1.55% in 2010 to 3.3% in 2014, with a slightly lower rate of 3.25% in 2014. We found significant positive spatial autocorrelations throughout 2010 to 2014 (Table 1), indicating that districts of similar risks were spatially clustered. Global autocorrelation was highest in 2010 (*I* = 0.388, *p* = 0.01) and lowest in 2012 (*I* = 0.095, *p* = 0.01). For easy interpretation, we presented the results of the local spatial autocorrelations as cluster maps based upon four categories: high-high, low-low, high-low, and low-high (Fig. 3). The high-high and the low-low associations indicate clustering of high risk (hot-spots) and low risks, respectively. Both the high-high and low-low associations indicate significant (*p* ≤ 0.05) clustering of similar risks or positive spatial autocorrelation. The low-high category indicates that high risk districts surround a low risk district, whereas the high-low category indicates that low risk districts surround a high risk district. These are indications of spatial outliers. High-high clustering dominated within the middle belt while low-low clustering dominated within the northern parts. Although we undertook no formal causal relationships since this is an exploratory study, a visual assessment of the local Moran’s *I* maps together with the ecological zones map of Ghana (Fig. 2) was worthwhile. We found that the high-high clusters extended within the semi-deciduous forest and the transitional ecological zones. The low-low clusters on the other hand were concentrated within the northern parts and mostly intersected with the Guinea and Sudan Savannah ecological zones. Few outliers were detected throughout the study period.

**Table 1:** Yearly incidence rates and global Moran’s *I*.

**Year**	**Cases**	**Rate (%)**	**Moran’s** ***I***	***p-value***
2010	381307	1.55	0.388	0.01
2011	505614	2.00	0.367	0.01
2012	663576	2.57	0.095	0.01
2013	875921	3.30	0.248	0.01
2014	884232	3.25	0.165	0.01

### Spatial structure and smoothing

**Figure 4:**
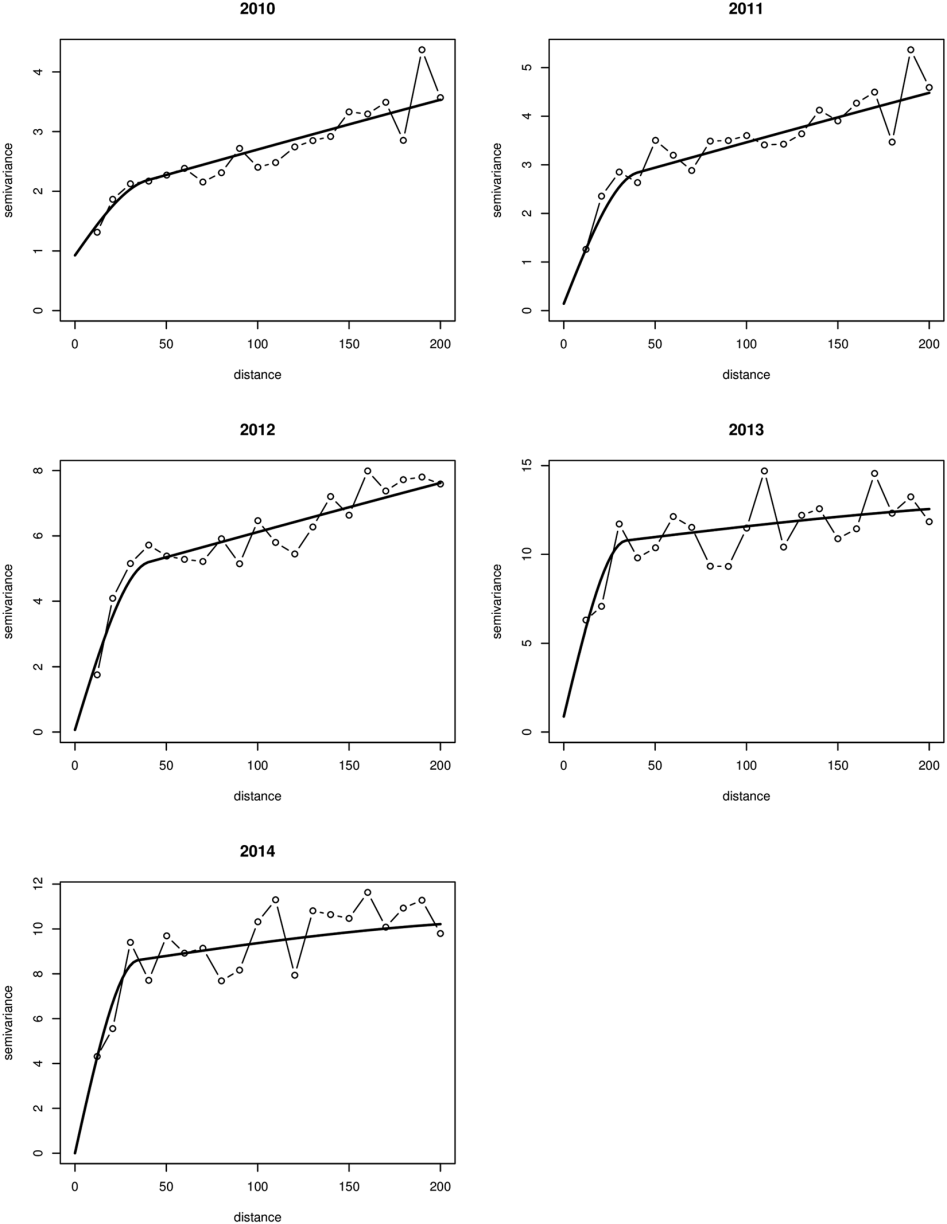
Nested variogram models of the risk for 2010 to 2014. This figure was created using R software (R Development Core Team 2013).

We computed experimental variograms for each year using 10 km lag distances for 20 lags and fitted nested spherical models (Fig. 4). At 10 km lags, there were enough (≥30) pairs of districts to obtain stable estimates of the variogram. Table 2 shows the parameters of the models fitted to the experimental variogram. All variograms exhibited two basic structures. The parameter *c*_0_ is the nugget variance which refers to spatially random variation. The variance parameter *c*_1_ refers to an estimate of the amount of spatially structured local (short range) variation within an average range of *ϕ*_1_. The variance parameter *c*_2_ refers to an estimate of the amount of spatially structured regional (large range) variation within an average range of *ϕ*_2_. The sum $sill={c}_{0}+{c}_{1}+{c}_{2}$ refers to the total variation, and *%c*_0_, *%c*_1_, *%c*_2_ refers to the proportion of the overall variation explained by *c*_0_, *c*_1_, *c*_2_, respectively. We found that the local spatial variations fell within the range ≈ 34–41 km. The regional spatial variation, however, showed widespread range values, ≈271–900 km, beyond the maximum lag distance used for estimation. For the years 2010 to 2012, ${\hat{\gamma }}_{{\rm{reg}}}(h)$ accounted for nearly 70% or more of the variation and ${\hat{\gamma }}_{{\rm{loc}}}(h)$ accounted for nearly 30% or less. Conversely, for rates in 2013 and 2014, ${\hat{\gamma }}_{{\rm{loc}}}(h)$ accounted for more than 70% whereas ${\hat{\gamma }}_{{\rm{reg}}}(h)$ accounted for nearly 25% or less. We also found an increasing trend of the variation accounted for by ${\hat{\gamma }}_{{\rm{loc}}}(h)$ from ≈ 13% in 2010 to ≈ 75% in 2014.

**Table 2:** Summary of the variogram models and parameters fitted to the experimental variograms.

**Year**	***c***_**0**_	***%c***_**0**_	***c***_**1**_	***ϕ***_**1**_ **(km)**	***%c***_**1**_	***c***_**2**_	***ϕ***_**2**_ **(km)**	***%c***_**2**_
2010	0.925	13.40	0.919	39.603	13.31	5.060	883.147	73.29
2011	0.141	1.62	2.277	40.399	26.14	6.293	900.055	72.24
2012	0.067	0.48	4.506	41.186	32.48	9.300	900.051	67.04
2013	0.876	6.73	9.466	34.177	72.70	2.678	314.030	20.57
2014	0.000	0.00	8.184	34.608	74.89	2.744	271.626	25.11

**Figure 5:**
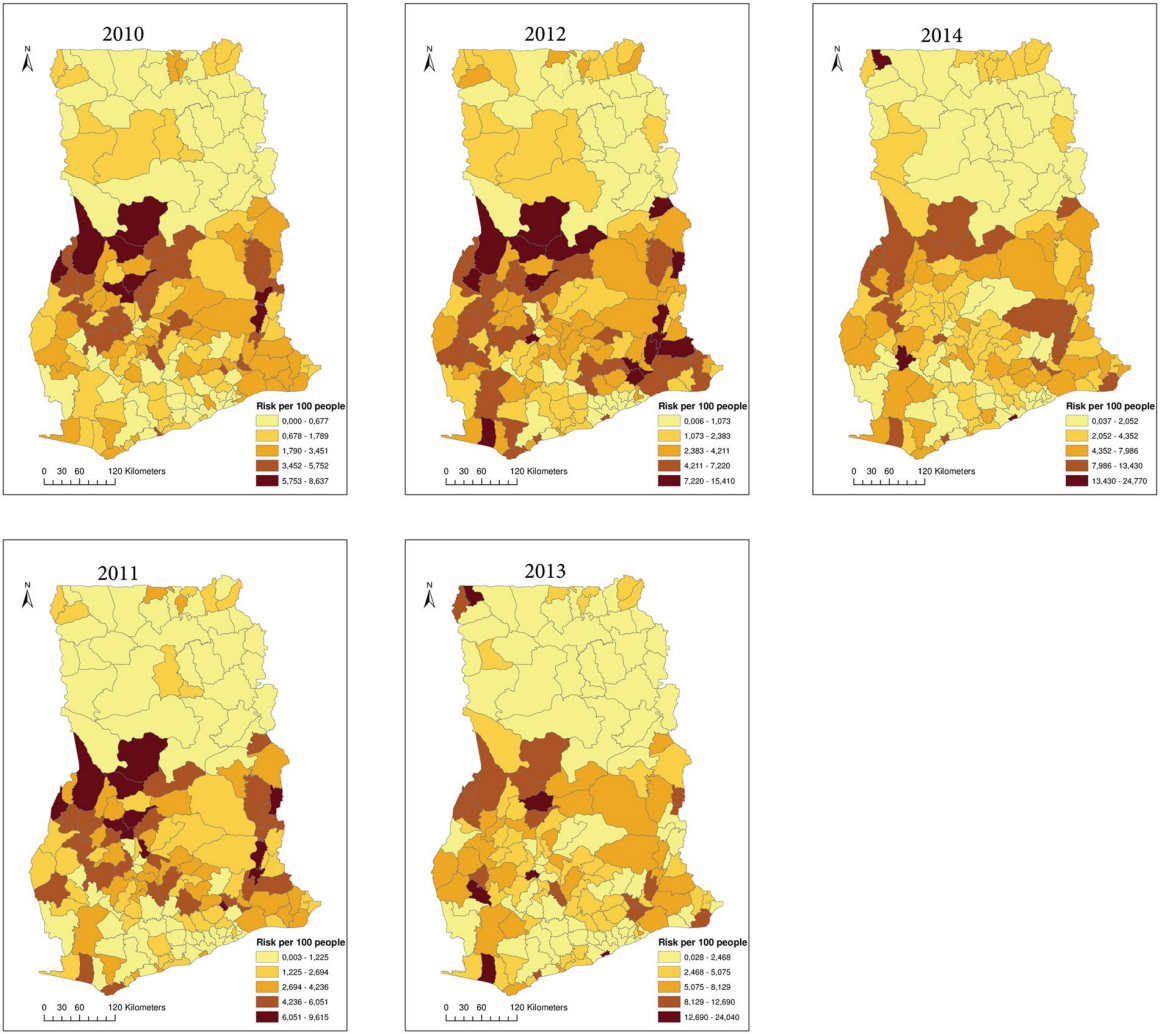
Maps of the smoothed rates after geostatistical filtering. This map was created using ArcGIS software (version 10.1, ESRI Inc. Redlands, CA, USA. https://www.esri.com/).

**Figure 6:**
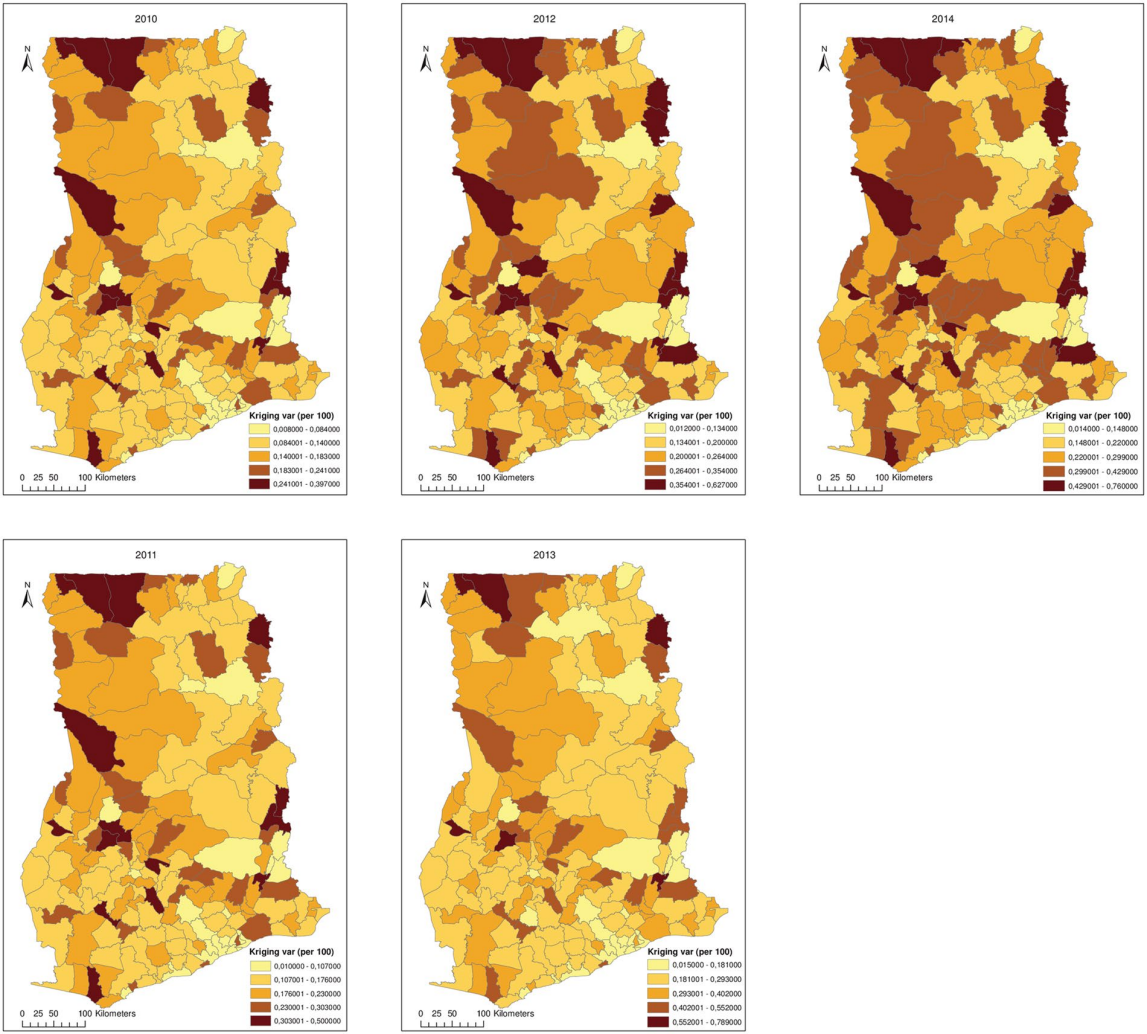
Maps of the Poisson kriging variance after geostatistical filtering. This map was created using ArcGIS software (version 10.1, ESRI Inc. Redlands, CA, USA. https://www.esri.com/).

**Figure 7:**
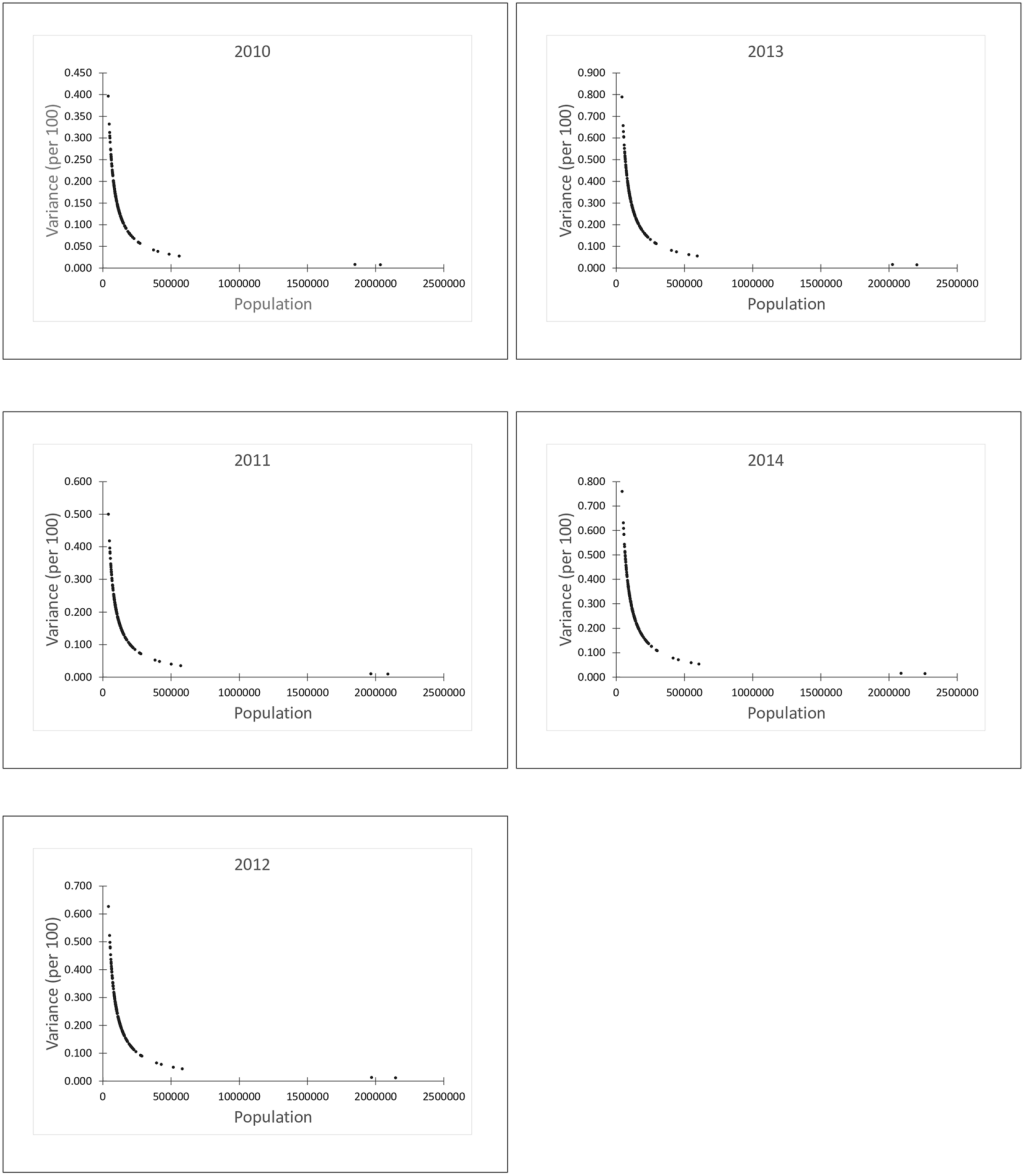
Graphs of variances accounted for against population after Poison kriging.

Maps of the smoothed rates and kriging variance after geostatistical filtering are shown in Figs 5 and 6, respectively. Both the smoothed rates and the variances show clustering of districts with similar estimates. From the smoothed maps, we found that high rates dominate within the middle belt, whereas low rates dominate within the northern parts. As was expected, we found reduced variation and considerable adjustments of rates for districts with smaller populations than districts with larger populations (Fig. 7).

## Discussion

We observed several noteworthy insights. We found evidence of global clustering of districts with comparable risks, suggesting the importance of spatially dependent phenomena modulating the spatial heterogeneity in the risk of intestinal worms infection. The observed global patterns imply that neighboring districts have similar underlying ecological and environmental risk factors that trigger intestinal worms infections^28^. The similarity, however, widely contrasted as local Moran’s *I* illuminated that the high-high values (hot-spots) dominated within the middle belt, whereas low-low values (low risk) within the northern part. One would, on the contrary, expect the hot-spots to occur mainly within the northern parts of Ghana where the socioeconomically less privilege are mostly found. This finding is an unexpected departure from other previous studies^20,45–47^, and suggests that interaction effects of environmental and socioeconomic risk factors that combine best to enhance infection could play a role. Although the study did not build a formal causal explanation model for the clustering patterns, a visual comparison of the patterns with the ecological zones of Ghana generates a working hypothesis that is worth testing in future research efforts. The patterns of the hot-spots are plausible since they widely intersected with the semi-deciduous forest and the transitional ecological zones. High precipitation, which is mostly associated with the semi-deciduous forest and the transitional zones of Ghana, has been found to increase the risk of intestinal parasites^19^. The specific effect on precipitation, however, has been attributed to specific quarters of the year^19,21^. This emphasizes the need for further studies to substantiate this augment in our study area. The low risks on the other hand widely intersect within the Guinea and Sudan Savannah ecological zones. These ecological zones are mostly flat with low precipitation, high temperature, and consist predominantly of grassland. These zones also have much drier soils with the highest land surface temperatures due to their proximity to the Sahel and the Sahara, and likely provide unfavorable environmental and ecological conditions for transmission. Some studies have associated low risk of intestinal worms infection to these unfavorable conditions. For instance^24^, associated low risk of helminth infection with high land surface temperature in Kenya. A study of the spatial distribution of helminth infection across sub-Saharan Africa associated extreme dry soils with the absence of hookworm infections^19^. The patterns of hot-spots and low risks are similar to findings from a study in Cote d’Ivoire, where low risk of schistosomiasis was found in Savannah ecological zones and high risk extended into Forest ecological zones^48^. In a developing country like Ghana, an alternative interpretation of the clustered patterns could be based on variations in the reporting systems. However, the consistency in these patterns throughout the study period suggests that they are less likely to have been caused by only variations in the reporting systems.

The Poisson variogram estimator accounted for the effects of population heterogeneities and revealed actual spatial structures that could otherwise have been obscured from the traditional variogram estimator^39,40^. In conjunction with the global and local statistics, nested variograms allowed us to identify the spatial distribution of intestinal parasites infection, showing that spatial variation occurs on two different scales. The average range of the local scale variation was 38 km, suggesting a strong correlation between neighboring districts. The average range of the small scale variation was less than the minimum distance of 68 km within which each district would have at least one adjoining neighbor. This suggests that most of the patterns of the small range structure only cover a district plus its adjoining neighbors. The range of the large-scale variation, ≈271–900 km, was larger than the maximum lag distance probably because of the dependency on ecological processes which operate with marked variation at regional scales larger than the average size of the districts and their higher-order adjoining neighbors. The short range parameters in the second structure of the variogram in 2013 and 2014 could be due to temporal changes in the regional scale ecological processes that affect infections.

Under the assumption that the unknown risk is a spatial stochastic process, Poisson kriging of the spatial risk had the advantage to correct for extreme rates due to small populations. The Poisson kriged risk maps indicated that intestinal parasites infections are spatially varied and widely distributed. High risk markedly persisted within the middle belt and low risks within the northern sector. This partly corroborates with data presented in a previous^23^ which found low hookworm egg counts (<1egg g^−1^) mostly in the northern parts and high hookworm egg counts (>30 eggs g^−1^) within central parts. Temporal changes in the spatial patterns over the years have been marginal, probably because the risk factors have generally remained static over time. Further studies to substantiate this argument will be valuable.

Our study still has some limitations. The first limitation relates the data. Data from the CHIM likely have recording gaps due to the voluntary reporting nature. Most reporting facilities (hospitals, clinics) lack diagnostic apparatus for proper biological confirmation of infection and hence rely on symptomatic diagnosis. However, we share the same opinion with Julian^49^ that imperfect information is likely more useful to intervention design than no information. Secondly, the morbidity data covered large heterogeneous districts. Although making inferences at district level was our interest, centroid based Poisson kriging makes an implicit assumption of homogenous population and morbidity distribution within districts. This is overly simplistic and could have affected our final smoothed maps. Further studies using rigorous statistical estimations are required to attenuate possible misspecification.

## Conclusions

This study demonstrated the use of spatial statistical methods such as cluster analysis and geostatistical smoothing to explore and elucidate the spatial patterns of district level intestinal parasites infections. Local and global Moran’s *I* estimated and mapped spatial clustering of intestinal parasites. Our findings regarding global Moran’s *I* indicated a non-random spatial distribution of internal parasites infection, and prompt for further studies to investigate and enumerate possible environmental and socioeconomic factors that could account for such patterns. Local Moran’s *I* cluster maps are essential for guiding public health officials to develop cost-effective control measures and could ensure that control programs are focused appropriately. In consequence of our findings, health professionals should pay more intervention attention to the hot-spots locations. Besides, the findings regarding the pattern of global and local autocorrelations are important steps in a process leading to a proper model for intestinal worms infection in the future. Finally, the study demonstrated the usefulness of geostatistics for filtering out noise caused by heterogeneous populations, which is important for low morbidities recorded in areas with low population sites. The geostatistical risk maps provided knowledge of the spatial distribution of intestinal parasites infections Ghana. We intend to investigate issues of spatial support further and varying population and morbidity distribution within districts in the future.
